# Functional connectivity of the nucleus basalis of Meynert in Lewy body dementia and Alzheimer’s disease

**DOI:** 10.1017/S1041610220003944

**Published:** 2021-01

**Authors:** Julia Schumacher, Alan J. Thomas, Luis R. Peraza, Michael Firbank, John T. O’Brien, John-Paul Taylor

**Affiliations:** 1 Translational and Clinical Research Institute, Faculty of Medical Sciences, Campus for Ageing and Vitality, Newcastle University, Newcastle upon Tyne, United Kingdom; 2 IXICO Plc, London, United Kingdom; 3 Department of Psychiatry, University of Cambridge School of Medicine, Cambridge, United Kingdom

**Keywords:** functional MRI, cholinergic system, dementia with Lewy bodies, Parkinson’s disease dementia

## Abstract

Cholinergic deficits are a hallmark of Alzheimer’s disease (AD) and Lewy body dementia (LBD). The nucleus basalis of Meynert (NBM) provides the major source of cortical cholinergic input; studying its functional connectivity might, therefore, provide a tool for probing the cholinergic system and its degeneration in neurodegenerative diseases. Forty-six LBD patients, 29 AD patients, and 31 healthy age-matched controls underwent resting-state functional magnetic resonance imaging (fMRI). A seed-based analysis was applied with seeds in the left and right NBM to assess functional connectivity between the NBM and the rest of the brain. We found a shift from anticorrelation in controls to positive correlations in LBD between the right/left NBM and clusters in right/left occipital cortex. Our results indicate that there is an imbalance in functional connectivity between the NBM and primary visual areas in LBD, which provides new insights into alterations within a part of the corticopetal cholinergic system that go beyond structural changes.

## Introduction

Lewy body dementia (LBD) includes dementia with Lewy bodies (DLB) and Parkinson’s disease dementia (PDD) and is the second most common form of neurodegenerative dementia after Alzheimer’s disease (AD) (McKeith *et al.*, 2017). Cholinergic deficits are a hallmark of LBD and AD, and have been shown to be related to overall cognitive impairment and the core LBD symptoms of visual hallucinations and cognitive fluctuations (Perry *et al.*, 1990).

The nucleus basalis of Meynert (NBM) within the basal forebrain provides the main source of cholinergic innervation to the cortex (Mesulam *et al.*, 1983). Given that over 90% of NBM neurons are cholinergic (Mesulam *et al.*, 1983), assessing functional connectivity between the NBM and the rest of the brain may provide a noninvasive tool for probing corticopetal cholinergic pathways and studying their degeneration in LBD and AD.

The aim of the present study was, therefore, to investigate NBM functional connectivity in a group of LBD patients, how NBM connectivity in these patients differs from AD and healthy controls, and whether abnormalities in NBM connectivity are related to cognitive impairment, visuoperceptual symptoms, and cognitive fluctuations in LBD.

## Methods

### Participants

The study involved 106 participants over 60 years of age: 46 were diagnosed with probable LBD (15 PDD and 31 DLB), 29 were diagnosed with probable AD, and 31 were age-matched healthy controls.

Patients were recruited from the local community-dwelling population who had been referred to old-age psychiatry and neurology services. Dementia diagnoses were performed independently by two experienced old-age psychiatrists according to consensus criteria for probable DLB (McKeith *et al.*, 2017), PDD (Emre *et al.*, 2007), and AD (McKhann *et al.*, 2011). The study was approved by the local ethics committee and written informed consent was obtained from all participants prior to study participation.

### Data acquisition

MR imaging was performed on a 3T Philips Intera Achieva scanner. Structural images were acquired with a magnetization-prepared rapid gradient-echo (MPRAGE) sequence, sagittal acquisition, echo time 4.6 ms, repetition time 8.3 ms, inversion time 1250 ms, flip angle = 8°, SENSE factor = 2, and in-plane field of view 240 × 240 mm^2^ with slice thickness 1.0 mm, yielding a voxel size of 1.0 × 1.0 × 1.0 mm^3^.

Resting-state scans were obtained with a gradient-echo echo-planar imaging sequence with 25 contiguous axial slices, 128 volumes, anterior–posterior acquisition, in-plane resolution = 2.0 × 2.0 mm, slice thickness = 6 mm, repetition time = 3000 ms, echo time = 40 ms, and field of view = 260 × 260 mm^2^. LBD patients who were taking dopaminergic medication were scanned in the motor ON state.

### MRI preprocessing

Preprocessing was the same as in (Schumacher *et al.*, 2018). Briefly, FMRI Expert Analysis Tool from FMRIB Software Library (FSL) (www.fmrib.ox.ac.uk/fsl) was used for motion correction, slice timing correction, and spatial smoothing with a 6 mm full width at half maximum Gaussian kernel. Participants were only included if the estimated motion parameters were below 2 mm translation and 2° rotation. Denoizing was performed using ICA-AROMA in FSL, followed by regression of CSF and white matter signals. Functional and structural images were coregistered using boundary-based registration in FSL and normalized to the standard Montreal Neurological Institute (MNI) template using Advanced Normalization Tools. Functional data were high-pass filtered with a cutoff of 150 s and resampled to a resolution of 4 × 4 × 4 mm^3^. Gray matter probability maps were obtained from FSL’s FMRIB’s Automated Segmentation Tool.

### Seed-based functional connectivity analysis

**Figure 1.**
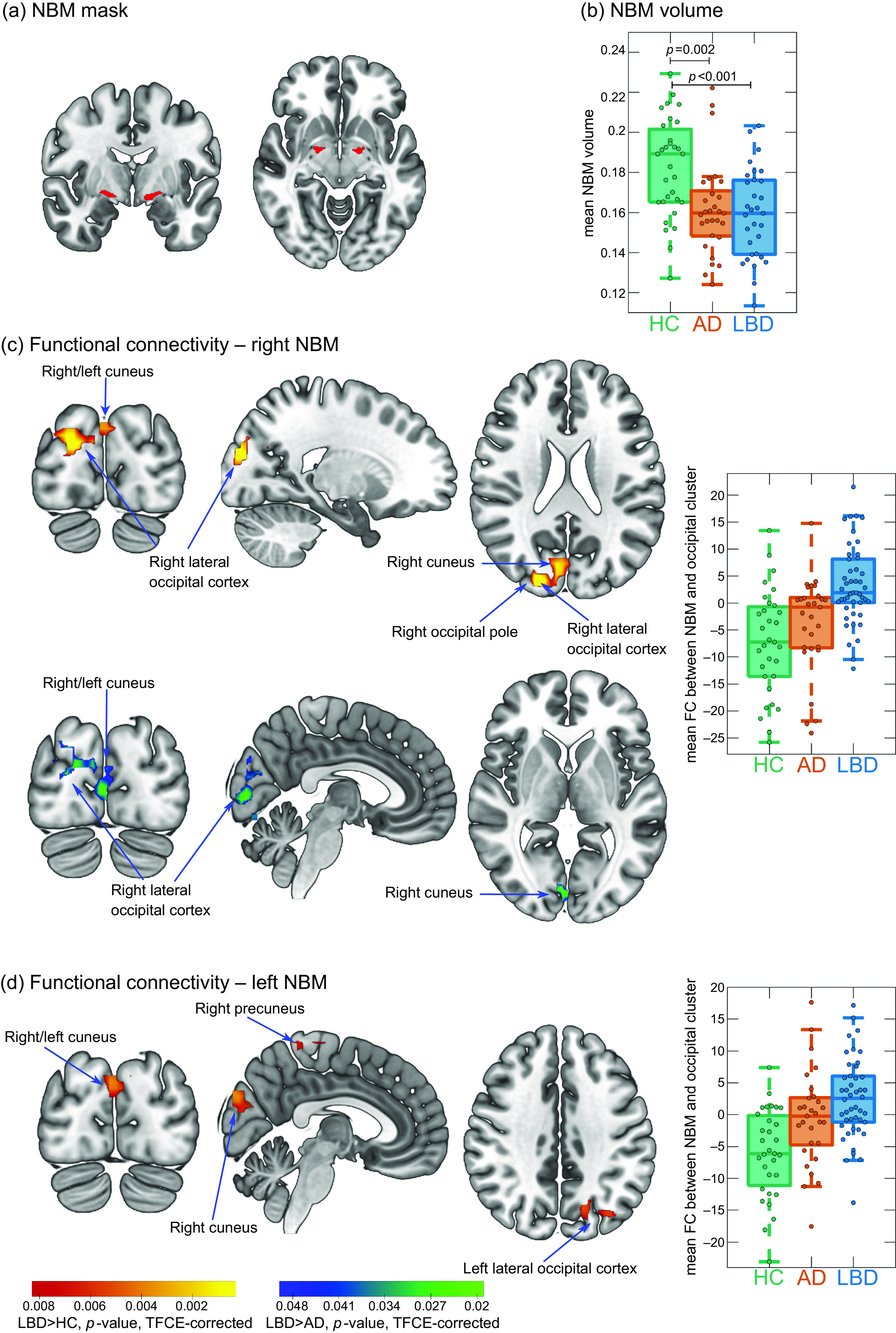
(a) NBM mask is used as a seed for the functional connectivity analysis. (b) Group comparison of NBM volume. (c and d) Clusters of significantly increased connectivity with the NBM in LBD compared to controls (red–yellow) and in LBD compared to AD (blue–green). Images are shown in the radiological convention, that is, the left side of the image corresponds to the right hemisphere. Boxplots show mean connectivity between the NBM and the occipital cluster, that is, *β* values from the dual regression. Positive values correspond to a positive association while negative values indicate an anticorrelation between the NBM seed time course and the time course of the occipital cluster. In each boxplot, the central line corresponds to the sample median, the upper and lower border of the box represent the 25th and 75th percentile, respectively, and the length of the whiskers is 1.5 times the interquartile range. AD, Alzheimer’s disease; FC, functional connectivity; HC, healthy controls; LBD, Lewy body dementia; NBM, nucleus basalis of Meynert; TFCE, threshold-free cluster enhancement.

The NBM was identified using a probabilistic anatomical map from the SPM Anatomy Toolbox which is based on microscopic delineations of 10 postmortem human brains (Zaborszky *et al.*, 2008). The NBM forms part of the basal forebrain, which consists of cholinergic cells that can be histologically defined as Ch1–Ch6 where Ch4 corresponds to the NBM (Mesulam *et al.*, 1983). Two seed regions containing the Ch4 subregions of right and left hemisphere, respectively, were created using the SPM Anatomy Toolbox (Figure 1a). The seed masks were downsampled to the resolution of the functional images using FLIRT in FSL and binarized (Supplementary Figure S1). Functional connectivity between right and left NBM seeds and the rest of the brain was determined for each participant using dual regression in FSL.

NBM volumes were estimated using the same NBM mask. Structural MR images were segmented into gray matter, white matter, and cerebrospinal fluid. The gray matter images were coregistered and normalized to MNI space using SPM’s DARTEL algorithm and modulated. Finally, images were smoothed with a 4 mm full width at half maximum Gaussian kernel. Gray matter volume within the NBM mask was calculated and normalized with respect to total intracranial volume using proportional normalization.

### Statistics

Group differences in NBM functional connectivity were assessed using FSL’s randomize tool with 10,000 permutations and family-wise error correction for multiple comparisons using threshold-free cluster enhancement (TFCE). Covariates for age, sex, and a voxel-wise covariate for gray matter density were included in the analysis. *p*-values were further corrected using Bonferroni correction across the six different group comparisons resulting in *α* = 0.05/6 = 0.0083; only clusters that exceeded this threshold were considered significant.

In an exploratory analysis, Spearman’s correlations between mean functional connectivity within significant clusters (from the comparison between LBD and controls) and clinical scores were tested for the Mini-Mental State Examination (MMSE) and Cambridge Cognitive Examination (CAMCOG) as measures of overall cognition, the Neuropsychiatric Inventory (NPI) hallucination subscale for visual hallucinations, the CAMCOG perception subscore for visuoperceptual impairment, and the Clinician Assessment of Fluctuations (CAF) score as a measure of cognitive fluctuation severity. Voxel-wise correlations with clinical scores inside significant clusters from the comparison between LBD and controls were assessed using a general linear model in FSL with the respective clinical score as covariate (including covariates of no interest for age and sex) and statistical significance was assessed using FSL’s randomize with TFCE correction.

NBM volume was compared between the groups using a univariate ANOVA and Spearman’s correlations between NBM volume and mean functional connectivity within significant clusters were calculated.

## Results

### Demographics

All three groups were similar in age and sex and the dementia groups showed similar overall cognitive impairment (Table 1). As expected, the LBD group was more impaired in terms of the core LBD symptoms compared to AD.

**Table 1.** Demographic and clinical variables, mean (standard deviation)

	HC (*n* = 31)	AD (*n* = 29)	LBD (*n* = 46)	Group differences
Male:female	22:9	20:9	34:12	χ^2^ = 0.23, *p* = 0.89^a^
Age	76.4 (7.2)	75.2 (8.6)	75.9 (6.9)	*F*(2,102) = 0.2, *p* = 0.82^b^
AChEI	–	26	40	χ^2^ = 0.01, *p* = 0.92^c^
PD meds	–	1	33	χ^2^ = 33.5, *p* < 0.001^c^
Duration	–	3.7 (1.7)^d^	3.2 (2.0)^e^	*U* = 455, *p* = 0.06^f^
MMSE	28.9 (1.1)	21.8 (3.8)	22.6 (3.9)	*t*_73_ = 0.9, *p* = 0.39^g^
UPDRS III	1.9 (2.8)	3.5 (4.0)	20.5 (9.9)	*t*_73_ = 8.8, *p* < 0.001^g^
CAF total	–	1.0 (2.5)^d^	5.5 (4.8)^h^	*t*_69_ = 4.5, *p* < 0.001^g^
NPI total	–	5.9 (5.5)^i^	17.2 (11.9)^e^	*t*_69_ = 4.6, *p* < 0.001^g^
NPI hall	–	0 (0)^j^	1.9 (1.9)^e^	*t*_68_ = 5.0, *p* < 0.001^g^

Abbreviations: AChEI, number of patients taking acetylcholinesterase inhibitors; AD, Alzheimer’s disease; CAF total, Clinician Assessment of Fluctuation total score; Duration, duration of cognitive symptoms in years; HC, healthy controls; LBD, Lewy body dementia; MMSE, Mini-Mental State Examination; PD meds, number of patients taking dopaminergic medication for the management of Parkinson’s disease symptoms; UPDRS III, Unified Parkinson’s Disease Rating Scale III (motor subsection); NPI, Neuropsychiatric Inventory; NPI hall, NPI hallucination subscore.

a χ^2^ test HC, AD, LBD.

b One-way ANOVA HC, AD, LBD.

c χ^2^ test AD, LBD.

d *N* = 28.

e *N* = 44.

f Mann–Whitney *U* test AD, LBD.

g Student’s *t*-test AD, LBD.

h *N* = 43.

i *N* = 27.

j *N* = 26.

### Functional connectivity

There was an increase in functional connectivity in the LBD group compared to controls between the right NBM and a cluster in right occipital pole, right lateral occipital cortex, and bilateral cuneal cortex (155 voxels). Similarly, for the left NBM, functional connectivity was increased in LBD compared to controls for a cluster in left lateral occipital cortex and bilateral cuneal cortex (116 voxels). For both right and left NBM, the clusters of increased connectivity with occipital cortices, in fact, showed a shift from anticorrelation in controls to positive correlations in LBD (Figure 1c and d). Furthermore, there was a small cluster of increased connectivity in LBD compared to controls between left NBM and the right precuneus (14 voxels).

For the right NBM, at the less conservative threshold of *p* < 0.05 (TFCE-corrected, but not corrected across the six group comparisons), connectivity was increased in LBD compared to AD in a cluster covering right cuneus, right lateral occipital cortex, and bilateral lingual gyrus (189 voxels, Figure 1c).

There were no differences in NBM functional connectivity between AD and controls (all *p* > 0.05, TFCE-corrected).

Comparing NBM functional connectivity between the LBD subgroups (i.e. DLB vs. PDD) did not reveal any significant differences.

NBM volume was significantly reduced in both AD and LBD compared to controls with no significant difference between the two dementia groups (Figure 1b). There was no significant correlation between mean functional connectivity in significant clusters and NBM volume (all *p* > 0.1).

### Clinical correlations

There were no significant correlations between NBM functional connectivity and any of the included clinical scores in the LBD group (all *p* > 0.1). Similarly, the voxel-wise regression analysis did not reveal any significant correlations with clinical scores.

## Discussion

In this study, we found a shift from negative to positive functional connectivity between the NBM and visual occipital areas in LBD patients compared to healthy controls and AD patients. This was symmetrically found for the right and left NBM and was not related to structural changes within the NBM.

Similar to the findings in our control group, previous studies have reported negative functional connectivity between the NBM and occipital areas in healthy participants (Li *et al.*, 2014). The results of the present study suggest that this normal anticorrelation is shifted toward a more positive synchronization between the NBM and visual areas in LBD. Given that the NBM is the major source of cholinergic innervation to the cortex, this altered connectivity profile might indicate an imbalance within the cholinergic system and a change in the cholinergic input that is provided to the occipital cortex. While the interpretation of negative correlations in functional magnetic resonance imaging (fMRI) functional connectivity can be challenging (see below), one possible interpretation of a shift from negative to positive correlations is a loss of inhibition. Indeed, a loss of inhibition of primary visual cortex has been reported in DLB patients before (Taylor *et al.*, 2016). Furthermore, Taylor *et al.* (2016) found an increase in BOLD activity in the primary visual cortex in DLB patients taking cholinesterase inhibitors compared to those patients not on this medication, indicating that the loss of inhibition of visual areas might be modulated by the cholinergic system. This hypothesis is supported by the results of the present study.

A similar shift from anticorrelation to positive correlations between the substantia innominata and parietal and occipital areas has previously been found in patients with Parkinson’s disease and mild cognitive impairment (Kim *et al.*, 2017). Furthermore, we found similar results in the DLB and PDD subgroups and we did not see any significant changes in AD patients compared to controls. Taken together, these results indicate that imbalances in functional connectivity between NBM and occipital cortex might be a feature that is specific to conditions characterized by alpha-synuclein pathology.

Even though previous studies have found a link between cholinergic system alterations and visual hallucinations in LBD (Perry *et al.*, 1990), we did not find an association between the extent of functional connectivity changes and the severity of visual hallucinations or visuoperceptual deficits. The observed functional alterations within the cholinergic system may create an environment that is permissive for the occurrence of symptoms such as visual hallucinations, possibly by a loss of inhibition of visual areas, but other factors may also be important in driving visual hallucinations.

Most previous studies of NBM functional connectivity have restricted their analyses to positive correlations because the interpretation and biological meaning of negative functional connectivity is still a subject of debate. While fMRI anticorrelations have been seen as an artifact of global signal regression (a preprocessing technique often used to remove global physiological noise), it has more recently been shown that they have a biological origin (Chai *et al.*, 2012). We did not include global signal regression in our preprocessing pipeline, but used an ICA-based denoizing approach instead. We would also argue that the symmetry of the cortical patterns in our findings is an indicator of their biological origin.

A limitation of this study is the relatively low resolution of the fMRI data, which is especially relevant given the size of the NBM. These results should, therefore, be interpreted with caution and should be replicated in independent cohorts with higher resolution data. The analyses would also benefit from being replicated in a multi-echo dataset, which has been shown to improve the signal-to-noise ratio, particularly in the basal forebrain (Markello *et al.*, 2018). Furthermore, due to the higher incidence rate of LBD in men and matching of the AD to the LBD group, the AD group comprised of more male participants, which might impact on the generalizability of the present findings in AD.

In conclusion, we showed that there is an imbalance within the connections between the NBM and the early visual system, which is specific to LBD and provides new insights into LBD-related imbalances within the cholinergic system that go beyond structural alterations.

## Supplementary material

For supplementary material accompanying this paper visit https://doi.org/10.1017/S1041610220003944. click here to view supplementary material
