# Supplementary material for: Functional connectivity of the nucleus basalis of Meynert in Lewy body dementia and Alzheimer’s disease
Source: Int Psychogeriatr. 2021 Jan 8;33(1):89–94. doi: 10.1017/S1041610220003944 (PMC8482375; doi:10.1017/S1041610220003944)
Supplement: Supplementary file 1 [file S1041610220003944sup001.docx]

Supplementary Material:
Functional connectivity of the nucleus basalis of Meynert in Lewy body dementia and Alzheimer’s disease

Julia Schumacher, Alan J. Thomas, Luis R. Peraza, Michael Firbank, John T. O’Brien, John-Paul Taylor

Correspondence: [julia.schumacher@newcastle.ac.uk](mailto:julia.schumacher@newcastle.ac.uk)

Content

1. Resampling of NBM mask
2. Voxel-based morphometry analysis

1. Resampling of NBM mask


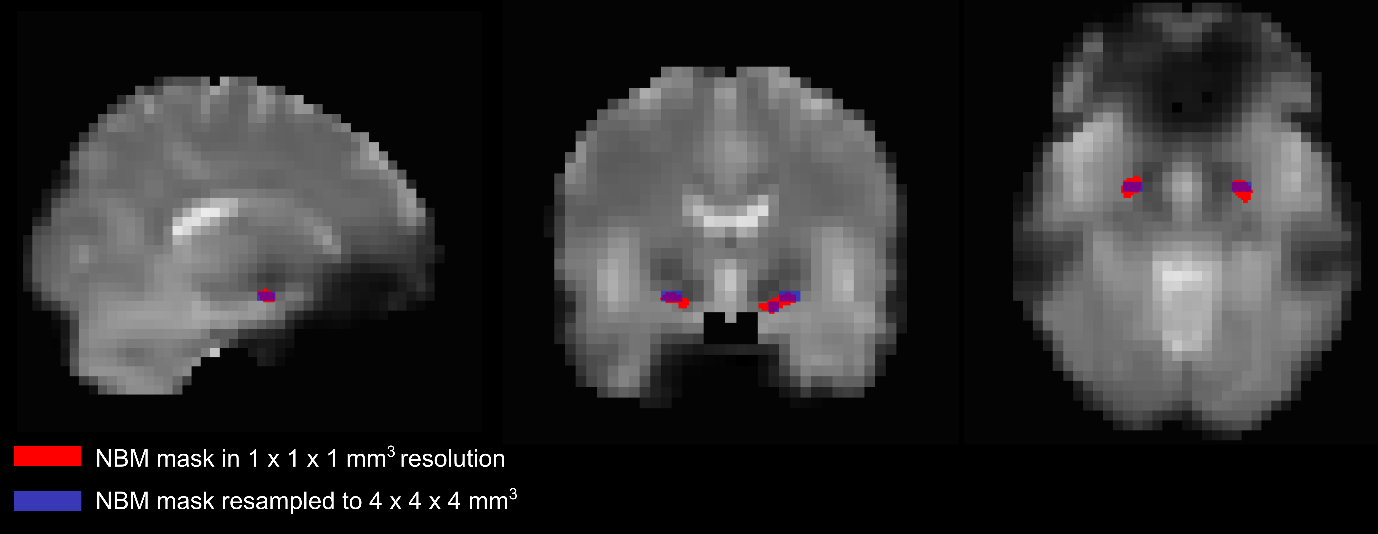


Supplementary Figure S1: The original NBM mask from the SPM Anatomy toolbox with a resolution of 1x1x1 mm^3^ is displayed in red, overlayed on an example subject’s fMRI data which have been normalised to MNI space. In blue, the NBM mask is displayed after resampling to the resolution of the fMRI data (4x4x4mm^3^) and thresholding at 0.4.

NBM, nucleus basalis of Meynert

2. Voxel-based morphometry analysis


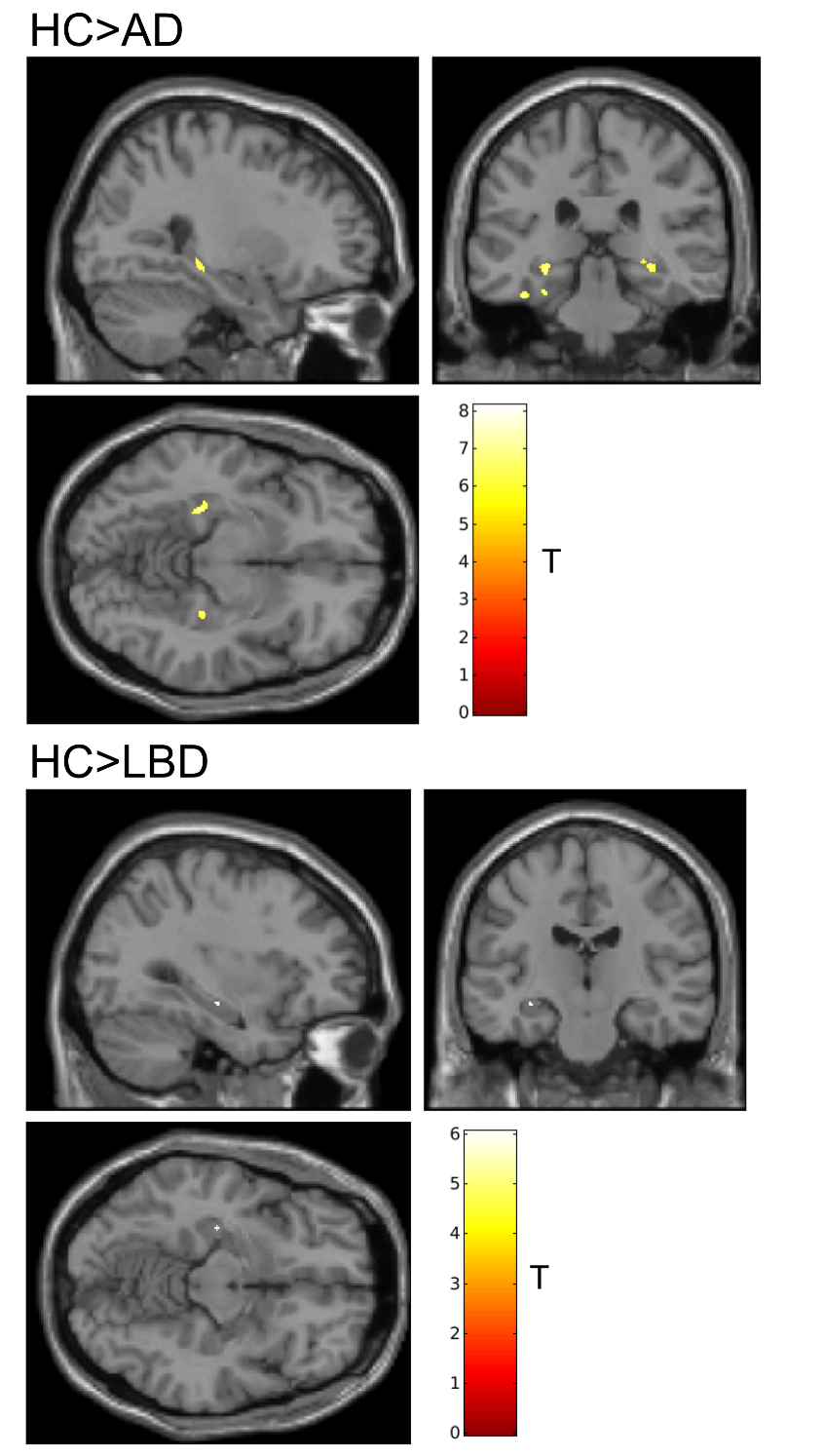


Supplementary Figure S2: Results from a voxel-based morphometry (VBM) analysis comparing whole-brain grey matter volume between the groups. The VBM analysis was performed in SPM12 using the DARTEL algorithm. Clusters are displayed that show a p-value<0.05, FWE-corrected. There were no significant differences between the AD and LBD groups.

AD, Alzheimer’s disease; HC, healthy controls; LBD, Lewy body dementia
